# Comparative analysis of anticoagulant influence on PMI estimation based on porcine blood metabolomics profile measured using GC-MS

**DOI:** 10.3389/fmolb.2024.1400622

**Published:** 2025-01-07

**Authors:** Patrycja Mojsak, Paulina Samczuk, Paulina Klimaszewska, Michal Burdukiewicz, Jaroslaw Chilimoniuk, Krystyna Grzesiak, Karolina Pietrowska, Justyna Ciborowska, Anna Niemcunowicz-Janica, Adam Kretowski, Michal Ciborowski, Michal Szeremeta

**Affiliations:** ^1^ Metabolomics and Proteomics Laboratory, Clinical Research Centre, Medical University of Bialystok, Bialystok, Poland; ^2^ Department of Genetic Research, Central Forensic Laboratory of the Police, Warsaw, Poland; ^3^ Institute of Biotechnology and Biomedicine, Autonomous University of Barcelona, Cerdanyola, Spain; ^4^ Faculty of Mathematics and Computer Science, University of Wroclaw, Wroclaw, Poland; ^5^ Chemical Research Laboratory, Forensic Laboratory of the Voivodeship Police Headquarters in Bialystok, Bialystok, Poland; ^6^ Department of Forensic Medicine, Medical University of Bialystok, Bialystok, Poland; ^7^ Department of Endocrinology, Diabetology and Internal Medicine, Medical University of Bialystok, Bialystok, Poland; ^8^ Department of Medical Biochemistry, Medical University of Bialystok, Bialystok, Poland

**Keywords:** post-mortem interval (PMI), animal model, blood biomarkers, metabolomics, GC-MS

## Abstract

**Introduction:**

Accurate post-mortem interval (PMI) estimation is essential in forensic investigations. Although various methods for PMI determination have been developed, only an approximate estimation is still achievable, and an accurate PMI indication is still challenging. Therefore, in this study, we employed gas chromatography-mass spectrometry (GC-MS)-based metabolomics to assess post-mortem changes in porcine blood samples collected with and without the addition of anticoagulant (EDTA). Our study aimed to identify metabolites dependent on the EDTA addition and time (taking into account the biodiversity of the studied organism) and those that are time−dependent but resistant to the addition of an anticoagulant.

**Methods:**

The experiment was performed on blood samples collected from 16 animals (domestic pig, breed: Polish Large White), 8 with and 8 without EDTA addition. The moment of death (time 0) and 15 additional time points (from 3 to 168 h after death) were selected to examine changes in metabolites’ levels in specific time intervals. We employed linear mixed models to study the relationship between metabolite intensities, time and presence of EDTA while accounting for the effect of individual pigs.

**Results and Discussion:**

We confirmed that the intensity of 16 metabolites (mainly amino acids) significantly depends on PMI and the presence of EDTA. However, the intensity of the ideal biomarker(s) for PMI estimation should be determined only by the time after death and not by external factors such as the presence of the anticoagulant agent. Thus, we identified 41 metabolites with time−dependent intensities that were not susceptible to EDTA presence. Finally, we assessed the performance of these metabolites in a PMI predictive model. Citraconic acid yielded one of the lowest errors in general PMI estimation (32.82 h). Moreover, similar errors were observed for samples with and without EDTA (33.32 h and 32.34 h, respectively). Although the small sample size and information leak in predictive modelling prevent drawing definite conclusions, citraconic acid shows potential as a robust PMI estimator.

## 1 Introduction

One of forensic medicine’s most essential and challenging tasks is estimating the post-mortem interval (PMI). This parameter is defined as the time elapsed since an individual’s death. Precise and accurate estimation of the PMI is remarkably important as it can help establish the timeline of events surrounding death (Gelderman et al., 2021). Forensic science currently offers many methods for PMI estimation, including various conventional methods, such as measurement of physical changes (Donaldson and Lamont, 2013; Ciaffi et al., 2018), biochemical components in different tissues and body fluids (Donaldson and Lamont, 2013), DNA or RNA degradation (Ciaffi et al., 2018), or analysis of rigour and livor mortis (Madea, 2016; Amendt et al., 2011). Nevertheless, only an approximate estimation can be derived from conventional methods, and an accurate PMI estimation is still difficult to obtain (Mathur and Agrawal, 2011). Thus, more reliable and accurate methods to estimate PMI are in demand.

Recent advances in the methods for estimating PMI have enabled us to determine post−mortem intervals more precisely (Mathur and Agrawal, 2011). Based on the literature review (Peyron et al., 2021; Szeremeta et al., 2021), it has been suggested that analysing the metabolomic composition of body fluids might provide a better tool for PMI estimation (Locci et al., 2023). The analysis of the post−mortem metabolomic changes in biological samples with mass spectrometry opens the way to develop new methods for PMI estimation. Different analytical platforms such as liquid chromatography−mass spectrometry (LC−MS) (Szeremeta et al., 2022; Zhang et al., 2022a; Zhang et al., 2022b; Pesko et al., 2020), gas chromatography−mass spectrometry GC−MS (Dai et al., 2019; Wu et al., 2018; Sato et al., 2015; Kaszynski et al., 2016), and nuclear magnetic resonance (NMR) (Fischer et al., 2014; Locci et al., 2021) have been used in PMI−oriented research. Among these, GC−MS is known to be one of the most efficient analytical platforms and, therefore, is well−established in metabolomics research (Mojsak et al., 2022). GC−MS has a distinct advantage over the other analytical platforms in terms of retention time, mass spectrometry reproducibility, and the availability of well−established commercial and in−house metabolite libraries (Beale et al., 2018). Due to all these advantages, this high−throughput detection technique was chosen to conduct the analyses.

Based on the literature review, it was confirmed that the application of GC−MS to the estimation of PMI is also gradually increasing (Dai et al., 2019; Wu et al., 2018; Sato et al., 2015; Kaszynski et al., 2016). Most often, the samples of post−mortem blood plasma (Donaldson and Lamont, 2013; Sato et al., 2015; Costa et al., 2015; Zelentsova et al., 2020) and vitreous humour (Zelentsova et al., 2020; Bonicelli et al., 2022) are used to search for potential PMI estimation markers; however, cerebrospinal, pericardial, and synovial fluids (Wenzlow et al., 2023) have also been considered. Due to the popularity of the use of blood in PMI estimation and its relatively easy collection at the crime scene, we selected this material to perform our study.

Blood trail age estimation could offer valuable information for reconstructing criminal events and their chronological assessment (Costa et al., 2015). Death results in extensive biochemical changes also in the blood due to the absence of circulating oxygen and the consequent cessation of aerobic respiration, altered enzymatic reactions, cessation of anabolic production of metabolites, cessation of active membrane transport and changes in the permeability of cells and diffusion of ions (Donaldson and Lamont, 2013). In these circumstances, using anticoagulants, such as EDTA, aids in suppressing the blood clotting mechanism to enable a longer examination time. On the other hand, there are many controversial studies regarding adding this anticoagulant (Bergmann et al., 2021). Based on UV-VIS analysis, Bergmann et al. confirmed that unnatural blood coagulation prevention is highly questionable when estimating bloodstain age, since the blood’s physical and chemical properties are altered (Bergmann et al., 2021). On the other hand, in most studies where blood samples were used to estimate PMI, it was anticoagulated with EDTA (Das et al., 2019; Sibbens et al., 2017; Wang et al., 2017). For this reason, we attempted to compare the metabolite profiles in both types of blood samples to check the impact of EDTA addition on the PMI estimation.

To the best of our knowledge, this is the first approach to estimate PMI in a porcine model using GC−MS−based metabolomics of plasma samples with or without EDTA addition. Considering the complementarity of GC−MS and LC−MS techniques in metabolomics studies (Zeki et al., 2020; Yumba-Mpanga et al., 2019), the present study is a valuable continuation of our previous research (Szeremeta et al., 2022). Apart from finding the differences between the metabolic profiles of blood samples with and without the addition of anticoagulants, we want to find universal metabolites that depend on the time since death, regardless of the addition of EDTA.

## 2 Materials and methods

### 2.1 Chemicals

O−methoxyamine hydrochloride, analytical grade of heptane and pyridine were supplied from Sigma−Aldrich (Steinheim, Germany). N,O−bis−(trimethylsilyl)− trifluoroacetamide (BSTFA) with 1% trimethylchlorosilane (TMCS) solution and acetonitrile (HPLC grade) was purchased from Thermo Fisher Scientific (Waltham, MA, USA). 4−nitrobenzoic acid (4−NBA) and stearic acid methyl ester (C18:0 methyl ester) were acquired as well from Sigma–Aldrich (Steinheim, Germany) and applied as internal standards (ISs). The 4−NBA (IS1) solution was prepared in acetonitrile, whereas methyl stearate in heptane (IS2). Two mixtures of standards for GC−MS, one containing grain fatty acid methyl esters (FAMEs) (C8:0−C22:1, n9) and another a mixture of n−alkanes (C8:C40), were obtained from Supelco (Bellefonte, PA, United States).

### 2.2 Sample collection and preparation

The experimental design used in this study was the same as described previously (Szeremeta et al., 2022). Sample preparation was carried out as previously described (Mojsak et al., 2022) with minor modifications. Briefly, an aliquot of 40 µL of plasma and 120 µL of cold acetonitrile containing the IS1 (25 ppm) were mixed for metabolites extraction. The mixture was vortexed for 2 min and centrifuged at 15,000 g for 10 min at 4°C. Finally, each sample’s supernatant (120 µL) was collected in a GC vial equipped with an insert and evaporated to complete dryness using a vacuum concentrator. All analysed samples were subjected to a two−step derivatisation process. First, methoxymation was performed by adding 30 µL of methoxylamine hydrochloride in pyridine solution (15 mg/mL) and then incubating at room temperature in the dark for 16 h. Following this, 30 µL of BSTFA with 1% TMCS was added to each sample and placed in the oven to react for 1 h at 70°C. At last, 90 µL of IS2 (10 ppm) was added as instrumental IS.

### 2.3 Quality control samples

To monitor the analytical variability and assess the reproducibility and repeatability of the methodology, quality control (QC) samples were used (Kirwan et al., 2022). The QC samples were prepared by pooling the study samples. Blank samples containing cold acetonitrile were used to detect the column’s contamination and the background noise produced during sample derivatisation, data processing and GC/MS analysis.

### 2.4 GC−MS−based untargeted metabolomics

Metabolic fingerprinting was performed using a 7890B gas chromatograph connected to a 7000D mass selective detector (Agilent Technologies, Palo Alto, CA, United States). A DB−5MS capillary column (30 m × 0.25 mm × 0.25 µm) was used for the chromatographic separation. One µL of each derivatised plasma sample was automatically injected at a split ratio of 1:10 using helium as a carrier gas with a 1 mL/min flow rate. The temperature of the injection was set to 250°C. The column oven temperature was maintained at 60°C for 1 min and then increased by 10°C/min to 320°C. The transfer line, ion source and quadrupole temperature were set at 280, 300°C and 150°C, respectively. Mass spectra were acquired under electron impact (EI) ionisation conditions using 70 eV in the mass range of m/z 50–600 using the default instrument scan rate. All samples (study samples, QCs and blanks) were analysed using the above-mentioned conditions.

### 2.5 Data processing

The deconvolution and identification were performed using Mass Hunter Quantitative Unknowns Analysis software (B.07.00, Agilent), alignment using Mass Profiler Professional software (version 13.0, Agilent) and peak integration using Mass Hunter Quantitative Analysis software (version B.07.00, Agilent). The identification was performed mainly based on the accurate mass and product ion spectrum matching against the in–house library of 100 authentic standards and Fiehn’s and NIST 14 libraries. Before the statistical analysis, peak areas were normalised by IS abundance to minimise the response variability from the instrument. Finally, data were filtered based on the coefficient of signal variation (CV) in QC samples, considering values lower than 30% as acceptable.

### 2.6 Statistical analyses

The exploratory analysis indicated high data variability between the timepoints and individual pigs. To adequately address this data structure, we employed the linear mixed model, where we considered the metabolite intensity to depend on both time (*t*) and the presence of EDTA. Moreover, we have included the impact of individual pigs as a random effect (*b*_ID_). Thus, our model considers the additional unknown variability affecting the intensity of a given metabolite over time (Equations 1, 2). We applied ANOVA to verify if the presence of EDTA is a significant variable in the model.

$$y=\beta_{0}+b_{\text{ID}}+\beta_{1}t+\beta_{2}\text{EDTA}$$ (1)

$$y=\beta_{0}+b_{\text{ID}}+\beta_{1}t$$ (2)

We predicted the measurement time considering the known metabolite intensity for each metabolite using the coefficients of model 2) to assess if the metabolite intensity could estimate PMI (Patterson and Thompson, 1971). Due to the small sample size, we performed a prediction on the samples used to fit the model, which caused the information leak. We evaluated the error of time prediction (in hours) for both models sensitive and those not sensitive to the presence of EDTA. We have used mean average error (MAE) as an error measure for samples with and without EDTA. Total mean average error (TMAE) was computed for all samples (with and without EDTA). Due to the limited dataset, we computed the performance on the same data we used to produce the model, leading to the information leak. The statistical analysis was performed in R 4.2.

## 3 Results

After data pretreatment (deconvolution, alignment, data normalisation and filtering), 126 entities were obtained, and 79 metabolites were annotated (Supplementary Table S1; Supplementary Materials), taking into account several derivatives from one metabolite [mainly for certain amino acids (AAs) and carbohydrates (Carbs)]. Finally, we chose 71 and 73 metabolites for statistical analysis, representing different analytical classes [mainly AAs, Carbs and fatty acids (FAs)], with RSD below 30% in plasma with and without EDTA addition, respectively.

**FIGURE 1:**
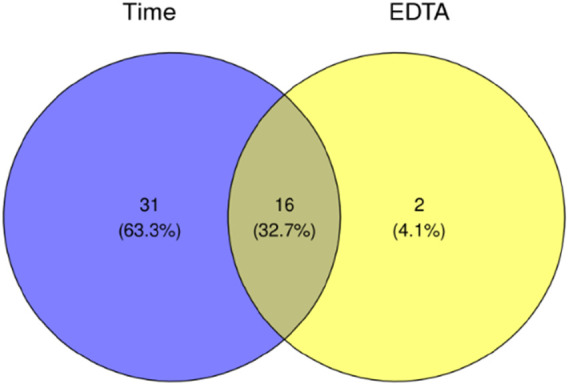
Ven diagram showing number for statistically significant metabolites.

Using ANOVA, we compared which of the two models significantly better captures the data structure. As model 2) is nested in model 1), we interpreted the ANOVA result as an indication of the presence of EDTA as a parameter necessary to describe the change of the metabolite intensity over time. After applying the Benjamini−Hochberg correction, we discovered 16 metabolites whose intensities depend on the EDTA presence and the time after death. Relationships between significant metabolites in EDTA−based and time−based tests were presented on the Venn diagram (Figure 1).

**FIGURE 2:**
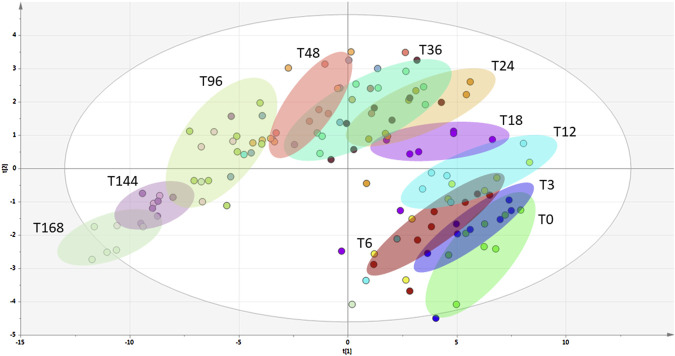
PLS-DA analysis of blood samples with EDTA collected at different post-mortal time-points.

The *p*−values for the 16 metabolites mentioned above are presented in Table 1, whilst their intensity−time plots showing the tested pigs’ biodiversity are presented in Supplementary Figure S1. Metabolites in blood containing EDTA are more stable than in blood without anticoagulant (see Figure S1). Additionally, we present the result of the PLS-DA analysis (Figure 2) performed for EDTA-treated serum metabolomics data at different post-mortal time points, illustrating the temporal variations in plasma composition.

**TABLE 1:** List of statistically significant metabolites dependent on time and EDTA addition with their *p*−value, adjusted *p*−value (Benjamini−Hochberg correction).

Group of metabolites	Metabolites	RT	HMDB ID	*p*−value	Adjusted *p*−value
AAs, peptides and analogues	Creatinine	13.6	HMDB00562	5.29E−14	5.52E−13
	Iminodiacetic acid	13.3	HMDB11753	4.01E−15	4.88E−14
	Isoleucine	10.05	HMDB00172	2.11E−07	1.40E−06
	Lysine	17.5	HMDB00182	0.000307	0.0014
	Ornithine	15.8	HMDB00214	3.29E−06	2.00E−05
	Phenylalanine	14.2	HMDB00159	2.85E−16	4.17E−15
	Threonine	11.3	HMDB00167	2.20E−28	5.36E−27
	Valine	7.2, 9.2	HMDB00883	5.28E−22	9.64E−21
	5−Oxoproline/pyroglutamic acid	13.1	HMDB00267	3.52E−12	2.85E−11
Alpha−keto acids and derivatives	Pyruvic acid	6.6	HMDB00243	6.47E−07	4.30E−06
Carb and Carb conjugates	Glucose	17.25, 17.4	HMDB00122	2.00E−35	7.31E−34
	Mannose	17.15, 17.45	HMDB00169	1.63E−40	1.19E−38
	1,5−anhydro−D−sorbitol	17	HMDB02712	3.54E−05	0.00017
Dicarboxylic acids and derivatives	Fumaric acid	10.9	HMDB00134	1.87E−07	1.36E−06
Glycerophosphates	Glycerol 1−phosphate	15.9	HMDB00126	5.00E−05	2.00E−04
Purines and purine derivatives	Hypoxanthine	16.5	HMDB00157	4.76E−06	2.67E−05

Reversing this reasoning, we identified 41 metabolites that depend significantly on time after death but do not show a significant dependence on the presence of EDTA (Supplementary Table S2). It must be emphasized that there was over−interpretation of the ANOVA result by drawing such conclusions. However, the potential usefulness of these metabolites as PMI estimators was demonstrated while keeping the same linear mixed model framework.

**FIGURE 3:**
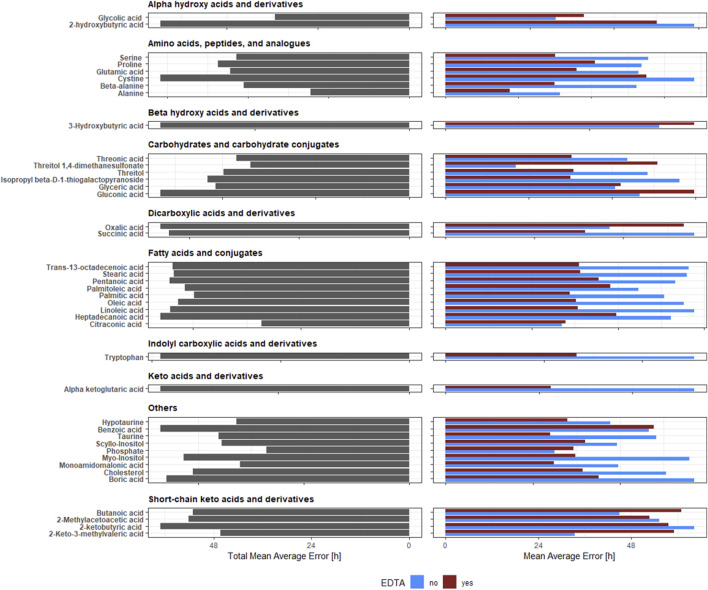
Total Mean Average Error [h] and Mean Average Error [h] of predictive models for PMI estimation with and without EDTA component. The height of the bar represents TMAE (left panel) and MAE (right panel). The bar’s colour (the right panel) represents the presence or absence of EDTA.

Two factors are vital for selecting the best metabolite for PMI estimation: low error (accuracy) and lack of sensitivity to blood clotting (universality). Among the considered metabolites, alanine, phosphate, and citraconic acid had the lowest TMAE (respectively, 29.29, 32.52, and 32.83 h) (Figure 3).

However, out of these three metabolites, only citraconic acid kept a comparably low MAE, independently of the presence or absence of EDTA (33.32 and 32.34 h, respectively). Both alanine and phosphate had drastically different MAE depending on the presence or absence of EDTA (alanine: 21.01 and 37.56; phosphate: 35.11 and 29.92 h, respectively).

**FIGURE 4:**
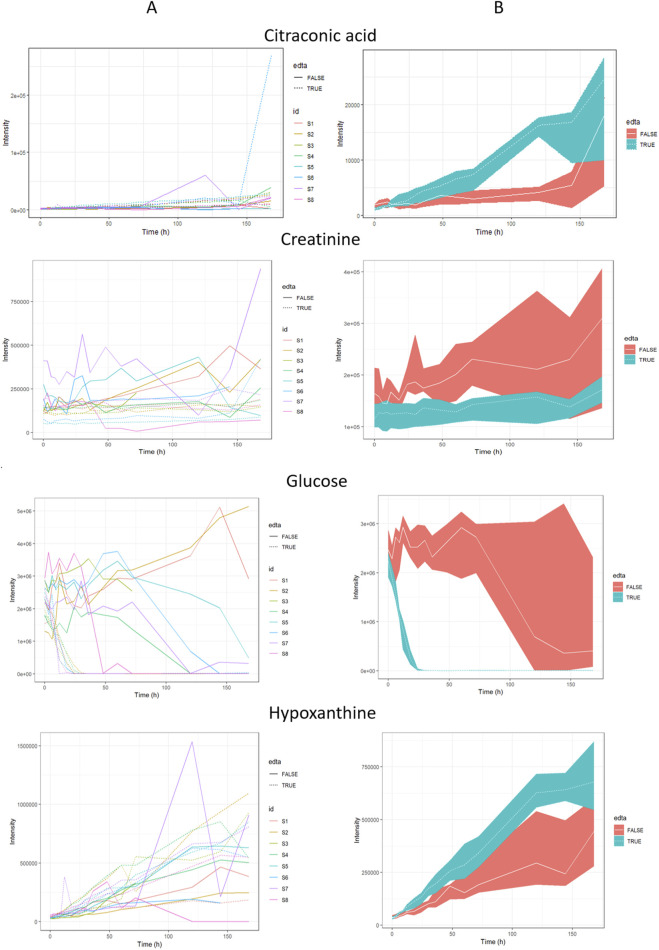
The most important metabolites for PMI estimation. Panel **(A)** - plots considering the biodiversity of the tested pigs; panel **(B)** - plots considering the median of all IDs for each time, metabolite, and EDTA, along with the first and third quartiles (Q1 and Q3).

Next, we investigated whether citraconic acid was the only metabolite that yielded equally accurate predictions regardless of EDTA presence. When considering the mean absolute difference in MAE, citraconic acid had the lowest score (0.98 h) (see Figure 4). Benzoic and glyceric acid were two other metabolites, with the mean absolute difference in TMAE being lower than 2 hours (1.37 and 1.50 h, respectively). However, they could not be reliably used to estimate PMI as their TMAE was very high (56.62 and 49.66 h, respectively).

This analysis does not exhaust the range of metabolites that could be useful for PMI estimation. Our analysis yielded 41 metabolites (Table S2) resistant to EDTA and with time−dependent intensity. However, their statistical significance was not followed up due to the low TMAE.

## 4 Discussion

Evaluation of the PMI has always been a major challenge for forensic pathologists (Wu et al., 2018). Despite many studies on PMI estimation and the development of various methods for PMI assessment over the years (Peyron et al., 2021; Laplace et al., 2021; Wilk et al., 2020; Wilk et al., 2021; Choi et al., 2019; Peng et al., 2020; Sangwan et al., 2021; De-Giorgio et al., 2021; Palacio et al., 2021), an accurate PMI indication, highly required in forensic medicine, is still complicated. The practical use of these methods in forensic science is impossible because most proposed approaches lack the reliability required to meet rigorous forensic standards (Locci et al., 2023).

Metabolomics analyses using mass spectrometry have recently gained popularity in forensic analysis (Kaszynski et al., 2016). This relatively new technology is often based on mass spectrometry, which allows the comprehensive study of low−molecular−weight metabolites (Lu et al., 2023). Especially during the agonal period, supravital reactions (occurring from death until cellular functions cease), leakage from degrading cells, and degradation of proteins affect the metabolomics profile. Therefore, analysing metabolites from biofluid samples can provide insights into the post−mortem changing biochemical environment (Donaldson and Lamont, 2013). Probably the most significant post-mortal changes in the blood metabolome are induced by cell membrane damage: the lack of ATP causes the dysfunction of intracellular Na+/K+ pumps, resulting in sodium accumulation inside the cells, cell lysis due to the osmotic pressure, and the leakage of the intracellular metabolites into the blood. The post−mortem metabolomic changes are also caused by the disruption of enzymatic cycles and microbial activity in the vascular system (Zelentsova et al., 2020).

Since the metabolomics approach to PMI estimation is still in its infancy, using animals as study subjects to determine the precise PMI is justified. Therefore, we built the PMI estimation model using porcine blood in the current study. Furthermore, ethical concerns make it considerably preferable to perform such a study with multiple time points on animal, not human blood (Matuszewski et al., 2020). Additionally, due to their structural and functional similarity to humans, pigs have been used as a model in biomedical research to evaluate PMI. It has been confirmed that pigs are a common human analogue in taphonomic studies (Connor et al., 2018) Researchers already confirmed that adding EDTA helps suppress the blood clotting mechanism, allowing the examination to be conducted over a longer period of time (Bergmann et al., 2021). On the other hand, this unnatural prevention of blood coagulation is highly questionable when estimating blood stain age since the blood’s physical and chemical properties are altered (Sharma and Kumar, 2018). Bergmann et al. confirmed that EDTA distorts blood spot ageing behaviour due to the prevention of coagulation (Bergmann et al., 2021), but based on different analyses conducted with UV/VIS spectra obtained for oxyhemoglobin, methemoglobin and hemichrome measurement. Until now, no study has reported using the metabolite profile analyses using GC−MS to estimate PMI in blood samples with or without the addition of an anticoagulant. For this reason, in this study, we examined the influence of EDTA on blood profiles over time to evaluate whether this effect occurs. Our results show that differences between the profiles of blood samples with and without EDTA addition were significant, which had already been proven in our previous study, performed using a complementary LC−MS technique (Szeremeta et al., 2022).

In the present study, we identified 16 metabolites with time-dependent intensities that were also affected by the presence of EDTA (see Figure S1). The best candidates for biomarkers for PMI estimation would be blood metabolites whose post−mortem intensity changes significantly, monotonously, relatively slowly, and with minimal data scattering (Sato et al., 2015). Most metabolites in plasma samples with EDTA (mainly AAs such as lysine, phenylalanine, threonine, valine, and pyroglutamic acid) met these criteria, with one exception for iminodiacetic acid, which met these criteria for both EDTA and non-EDTA samples. The intensity of AAs increases with time after death, and the most plausible explanation for this is the lack of energy. The cells began to break down proteins for energy and to combat bacterial spoilage, which led to the rapid degradation of a large number of proteins. It is worth noting that the increase in AAs was gradual rather than sudden, and the most likely explanation for this is that at the moment of death, not all the cells died, and within a certain time after death, some cells remained metabolically active (Wu et al., 2018).

Based on the graphs presented in Figure S1, it can be noticed that metabolites in blood with EDTA are more stable than in blood without anticoagulant addition. We observed larger data scattering in plasma samples without EDTA addition, especially at further times after death. Multiple concurrent biological processes induced by time may contribute to alterations in the post-mortem metabolome of blood samples untreated with EDTA (Go et al., 2019). Another critical difference in the change of the intensity of metabolites in the blood with the addition of EDTA is the rapid decrease in glucose intensity during the first day after death, with minimal data scattering (see Figure 4). The rapid decrease of glucose after death may also depend on post-mortem anaerobic glycolysis and the use of glucose by bacteria. After death, glycogen is used by skeletal muscles as a carbohydrate source for glycolytic substrate production, which generates ATP and lactate (Chauhan et al., 2019; Chauhan and England, 2018). In our study, we observed an increase in the intensity of lactic acid after death in two types of plasma samples. Lactate is not only produced during glycolysis but is also formed due to autolysis and bacterial catabolism. Several researchers have studied the correlation between lactic acid concentration and PMI (Mihailovic et al., 2011; Keltanen et al., 2015). The remaining compounds associated with EDTA include hypoxanthine and creatinine; the intensity of both metabolites increases with time (see Figure 4). Many previous publications have confirmed the use of these metabolites (i.e., creatinine or hypoxanthine) for PMI estimation (Peyron et al., 2021; Szeremeta et al., 2022; Dai et al., 2019; Sato et al., 2015; Kaszynski et al., 2016); and our research provides additional confirmation. Hypoxanthine concentration in the blood rises after death because it is an ATP−breakdown product that increases its concentration in situations of oxygen limitation and is available as a co−factor for xanthine oxidase. Changes observed for hypoxanthine correspond to breaking the tricarboxylic acid cycle and purine catabolism in an oxygen−deficient biological medium (Hira et al., 2014). Zelentsova et al. conducted a PMI study with rabbits and suggested that hypoxanthine and creatinine may have a strong potential as a PMI biomarker (Zelentsova et al., 2020), which was also confirmed in this study. After death, creatinine levels in the blood increase due to the cessation of kidney function and the breakdown of muscle tissue. Without renal filtration, creatinine accumulates in the bloodstream (Nishida et al., 2015). Measurement (in the vitreous humor) of hypoxanthine with potassium (Rognum et al., 2016; Madea and Rödig, 2006) and also urea (Cordeiro et al., 2019), has been reported useful for time since death estimation.

We have also indicated 43 metabolites whose intensities correlate with PMI and are not susceptible to EDTA presence. Using coefficients of the fitted models, we used intensities of these metabolites to estimate PMI (Figure 3). No metabolites with TMAE that were lower than 24 h, which severely hinder their usage in the case of estimating very short PMIs were identified. Moreover, we observed a much higher value of MAE for samples with EDTA than those without anticoagulant. Only citraconic acid could be characterised by the low TMAE (29.29 h) and comparable MAE regardless of the EDTA presence (33.32 h with EDTA and 32.34 without EDTA). Shen et al. showed that citraconic acid could be used to predict pork quality (Shen et al., 2022). This metabolite belongs to the dicarboxylic acid family, formed from the breakdown of citric acid. Citrate content, mainly in the bone, was confirmed as a metabolite associated with PMI (Wilson and Christensen, 2017; Brown et al., 2018).

However, it should be noted that the predictive model employed in this study suffers from the information leak, and the error estimate shown has to be overly optimistic. The precise assessment of its accuracy requires future research, where the sample size would allow for model validation using at least a leave−one−out setting. It was speculated that citraconic acid could aid in determining PMI.

Despite metabolomics being fraught with a high risk of large error (Zhang et al., 2023), findings show that this approach can be a powerful tool for predicting PMI (Aljeaid, 2024) The literature review confirmed that the metabolomic profile of the vitreous humour determined with NMR could predict PMI better than measuring potassium concentration, which was, until now, the best option for PMI estimation. It is crucial to bear in mind that the use of potassium concentration in PMI estimation is fraught with error in the range of 6.9 h for PMI <24 h, 7.4 h for PMI between 24 and 48 h, and 10.3 h for PMI >48 h (Locci et al., 2023).

## 5 Conclusion

Our preliminary study shows large differences between the two types of blood-originated samples. Metabolites in blood, with the addition of EDTA, maintain much better stability and are subject to much less data scattering. Adding EDTA helps suppress the blood clotting mechanism, providing a longer time to perform the examination. However, metabolites in blood behave more significantly without the addition of an anticoagulant. We observe a considerable dispersion of results in blood samples, especially at later times. Possible reasons might be multiple coexisting biological processes induced by time that affect metabolome post-mortem in blood samples untreated with EDTA.

Our analysis indicated only one metabolite (citraconic acid) suitable for PMI estimation, especially for PMIs longer than 1 day. However, it should be pointed out that this study had some limitations. First, the experiment was performed under constant environmental conditions, and further work should be directed to study the influence of environmental factors. Secondly, the study was conducted on animal material. Humans have a more complicated biological background and living habits than experimental animals. The application of the results of this study to the practical forensic investigation of human corpses needs further study.

## Data Availability

The raw data supporting the conclusions of this article will be made available by the authors, without undue reservation.

## References

[B1] Aljeaid R. (2024). Application of metabolomics and machine learning for the prediction of postmortem interval. Cureus 16 (11), e74161. 10.7759/cureus.74161 39575351 PMC11580817

[B2] Amendt J. Richards C. S. Campobasso C. P. Zehner R. Hall M. J. (2011). Forensic entomology: applications and limitations. Forensic Sci. Med. Pathol. 7 (4), 379–392. 10.1007/s12024-010-9209-2 21213072

[B3] Beale D. J. Pinu F. R. Kouremenos K. A. Poojary M. M. Narayana V. K. Boughton B. A. (2018). Review of recent developments in GC-MS approaches to metabolomics-based research. Metabolomics 14 (11), 152. 10.1007/s11306-018-1449-2 30830421

[B4] Bergmann T. Leberecht C. Labudde D. (2021). Analysis of the influence of EDTA-treated reference samples on forensic bloodstain age estimation. Forensic Sci. Int. 325, 110876. 10.1016/j.forsciint.2021.110876 34216943

[B5] Bonicelli A. Mickleburgh H. L. Chighine A. Locci E. Wescott D. J. Procopio N. (2022). The 'ForensOMICS' approach for postmortem interval estimation from human bone by integrating metabolomics, lipidomics, and proteomics. Elife 11, e83658. 10.7554/eLife.83658 36583441 PMC9803353

[B6] Brown M. A. Bunch A. W. Froome C. Gerling R. Hennessy S. Ellison J. (2018). Citrate content of bone as a measure of postmortem interval: an external validation study. J. Forensic Sci. 63 (5), 1479–1485. 10.1111/1556-4029.13716 29278649

[B7] Chauhan M. N. LeMaster M. N. Clark D. L. Foster M. K. Miller C. E. England E. M. (2019). Glycolysis and pH decline terminate prematurely in oxidative muscles despite the presence of excess glycogen. Meat Muscle Biol. 3 (1). 10.22175/mmb2019.02.0006

[B8] Chauhan S. S. England E. M. (2018). Postmortem glycolysis and glycogenolysis: insights from species comparisons. Meat Sci. 144, 118–126. 10.1016/j.meatsci.2018.06.021 29960720

[B9] Choi K. M. Zissler A. Kim E. Ehrenfellner B. Cho E. Lee S. I. (2019). Postmortem proteomics to discover biomarkers for forensic PMI estimation. Int. J. Leg. Med. 133 (3), 899–908. 10.1007/s00414-019-02011-6 PMC6469664 30864069

[B10] Ciaffi R. Feola A. Perfetti E. Manciocchi S. Potenza S. Marella G. (2018). Overview on the estimation of post mortem interval in forensic anthropology: review of the literature and practical experience. Romanian J. Leg. Med. 26, 403–411. 10.4323/rjlm.2018.403

[B11] Connor M. Baigent C. Hansen E. S. (2018). Testing the use of pigs as human proxies in decomposition studies. J. Forensic Sci. 63 (5), 1350–1355. 10.1111/1556-4029.13727 29284073

[B12] Cordeiro C. Ordóñez-Mayán L. Lendoiro E. Febrero-Bande M. Vieira D. N. Muñoz-Barús J. I. (2019). A reliable method for estimating the postmortem interval from the biochemistry of the vitreous humor, temperature and body weight. Forensic Sci. Int. 295, 157–168. 10.1016/j.forsciint.2018.12.007 30611119

[B13] Costa I. Carvalho F. Magalhães T. Guedes de Pinho P. Silvestre R. Dinis-Oliveira R. J. (2015). Promising blood-derived biomarkers for estimation of the postmortem interval. Toxicol. Res. 4 (6), 1443–1452. 10.1039/c5tx00209e PMC6062304 30102300

[B14] Dai X. Fan F. Ye Y. Lu X. Chen F. Wu Z. (2019). An experimental study on investigating the postmortem interval in dichlorvos poisoned rats by GC/MS-based metabolomics. Leg. Med. (Tokyo) 36, 28–36. 10.1016/j.legalmed.2018.10.002 30326392

[B15] Das S. Panda S. Acharya A. Mishra U. K. Kundu A. K. Mohanty B. (2019). Postmortem blood and tissue changes for estimation of time of death. Int. J. Curr. Microbiol. Appl. Sci. 8, 43–53. 10.20546/ijcmas.2019.809.007

[B16] De-Giorgio F. Ciasca G. Fecondo G. Mazzini A. De Spirito M. Pascali V. L. (2021). Estimation of the time of death by measuring the variation of lateral cerebral ventricle volume and cerebrospinal fluid radiodensity using postmortem computed tomography. Int. J. Leg. Med. 135 (6), 2615–2623. 10.1007/s00414-021-02698-6 PMC8523388 34562107

[B17] Donaldson A. E. Lamont I. L. (2013). Biochemistry changes that occur after death: potential markers for determining post-mortem interval. PLoS One 8 (11), e82011. 10.1371/journal.pone.0082011 24278469 PMC3836773

[B18] Fischer K. Kettunen J. Würtz P. Haller T. Havulinna A. S. Kangas A. J. (2014). Biomarker profiling by nuclear magnetic resonance spectroscopy for the prediction of all-cause mortality: an observational study of 17,345 persons. PLoS Med. 11 (2), e1001606. 10.1371/journal.pmed.1001606 24586121 PMC3934819

[B19] Gelderman T. Stigter E. Krap T. Amendt J. Duijst W. (2021). The time of death in Dutch court; using the Daubert criteria to evaluate methods to estimate the PMI used in court. Leg. Med. (Tokyo) 53, 101970. 10.1016/j.legalmed.2021.101970 34601451

[B20] Go A. Shim G. Park J. Hwang J. Nam M. Jeong H. (2019). Analysis of hypoxanthine and lactic acid levels in vitreous humor for the estimation of post-mortem interval (PMI) using LC-MS/MS. Forensic Sci. Int. 299, 135–141. 10.1016/j.forsciint.2019.03.024 31003185

[B21] Hira H. S. Samal P. Kaur A. Kapoor S. (2014). Plasma level of hypoxanthine/xanthine as markers of oxidative stress with different stages of obstructive sleep apnea syndrome. Ann. Saudi Med. 34 (4), 308–313. 10.5144/0256-4947.2014.308 25811203 PMC6152570

[B22] Kaszynski R. H. Nishiumi S. Azuma T. Yoshida M. Kondo T. Takahashi M. (2016). Postmortem interval estimation: a novel approach utilizing gas chromatography/mass spectrometry-based biochemical profiling. Anal. Bioanal. Chem. 408 (12), 3103–3112. 10.1007/s00216-016-9355-9 26931122

[B23] Keltanen T. Nenonen T. Ketola R. A. Ojanperä I. Sajantila A. Lindroos K. (2015). Post-mortem analysis of lactate concentration in diabetics and metformin poisonings. Int. J. Leg. Med. 129 (6), 1225–1231. 10.1007/s00414-015-1256-5 26459058

[B24] Kirwan J. A. Gika H. Beger R. D. Bearden D. Dunn W. B. Goodacre R. (2022). Quality assurance and quality control reporting in untargeted metabolic phenotyping: mQACC recommendations for analytical quality management. Metabolomics 18 (9), 70. 10.1007/s11306-022-01926-3 36029375 PMC9420093

[B25] Laplace K. Baccino E. Peyron P. A. (2021). Estimation of the time since death based on body cooling: a comparative study of four temperature-based methods. Int. J. Leg. Med. 135 (6), 2479–2487. 10.1007/s00414-021-02635-7 34148133

[B26] Locci E. Stocchero M. Gottardo R. Chighine A. De-Giorgio F. Ferino G. (2023). PMI estimation through metabolomics and potassium analysis on animal vitreous humour. Int. J. Leg. Med. 137 (3), 887–895. 10.1007/s00414-023-02975-6 PMC10085955 36799966

[B27] Locci E. Stocchero M. Gottardo R. De-Giorgio F. Demontis R. Nioi M. (2021). Comparative use of aqueous humour ^1^H NMR metabolomics and potassium concentration for PMI estimation in an animal model. Int. J. Leg. Med. 135 (3), 845–852. 10.1007/s00414-020-02468-w PMC8036180 33219398

[B28] Lu X. J. Li J. Wei X. Li N. Dang L. H. An G. S. (2023). A novel method for determining postmortem interval based on the metabolomics of multiple organs combined with ensemble learning techniques. Int. J. Leg. Med. 137 (1), 237–249. 10.1007/s00414-022-02844-8 35661238

[B29] Madea B. (2016). Methods for determining time of death. Forensic Sci. Med. Pathol. 12 (4), 451–485. 10.1007/s12024-016-9776-y 27259559

[B30] Madea B. Rödig A. (2006). Time of death dependent criteria in vitreous humor—accuracy of estimating the time since death. Forensic Sci. Int. 164 (2), 87–92. 10.1016/j.forsciint.2005.12.002 16439082

[B31] Mathur A. Agrawal Y. (2011). An overview of methods used for estimation of time since death. Aust. J. Forensic Sci. 43, 275–285. 10.1080/00450618.2011.568970

[B32] Matuszewski S. Hall M. J. R. Moreau G. Schoenly K. G. Tarone A. M. Villet M. H. (2020). Pigs vs people: the use of pigs as analogues for humans in forensic entomology and taphonomy research. Int. J. Leg. Med. 134 (2), 793–810. 10.1007/s00414-019-02074-5 PMC7044136 31209558

[B33] Mihailovic Z. Atanasijevic T. Popovic V. Milosevic M. B. (2011). Could lactates in vitreous humour be used to estimate the time since death? Med. Sci. Law 51 (3), 156–160. 10.1258/msl.2011.010124 21905571

[B34] Mojsak P. Maliszewska K. Klimaszewska P. Miniewska K. Godzien J. Sieminska J. (2022). Optimization of a GC-MS method for the profiling of microbiota-dependent metabolites in blood samples: an application to type 2 diabetes and prediabetes. Front. Mol. Biosci. 9, 982672. 10.3389/fmolb.2022.982672 36213115 PMC9538375

[B35] Nishida A. Funaki H. Kobayashi M. Tanaka Y. Akasaka Y. Kubo T. (2015). Blood creatinine level in postmortem cases. Sci. Justice 55 (3), 195–199. 10.1016/j.scijus.2014.12.005 25934372

[B36] Palacio C. Gottardo R. Cirielli V. Musile G. Agard Y. Bortolotti F. (2021). Simultaneous analysis of potassium and ammonium ions in the vitreous humour by capillary electrophoresis and their integrated use to infer the post mortem interval (PMI). Med. Sci. Law 61 (1_Suppl. l), 96–104. 10.1177/0025802420934239 32588729

[B37] Patterson H. D. Thompson R. (1971). Recovery of inter-block information when block sizes are unequal. Biometrika 58 (3), 545–554. 10.1093/biomet/58.3.545

[B38] Peng D. Lv M. Li Z. Tian H. Qu S. Jin B. (2020). Postmortem interval determination using mRNA markers and DNA normalization. Int. J. Leg. Med. 134 (1), 149–157. 10.1007/s00414-019-02199-7 31773316

[B39] Pesko B. K. Weidt S. McLaughlin M. Wescott D. J. Torrance H. Burgess K. (2020). Postmortomics: the potential of untargeted metabolomics to highlight markers for time since death. OMICS 24 (11), 649–659. 10.1089/omi.2020.0084 33095683 PMC7687049

[B40] Peyron P. A. Hirtz C. Baccino E. Ginestet N. Tiers L. Martinez A. Y. (2021). Tau protein in cerebrospinal fluid: a novel biomarker of the time of death? Int. J. Leg. Med. 135 (5), 2081–2089. 10.1007/s00414-021-02558-3 33740116

[B41] Rognum T. O. Holmen S. Musse M. A. Dahlberg P. S. Stray-Pedersen A. Saugstad O. D. (2016). Estimation of time since death by vitreous humor hypoxanthine, potassium, and ambient temperature. Forensic Sci. Int. 262, 160–165. 10.1016/j.forsciint.2016.03.001 26994446

[B42] Sangwan A. Singh S. P. Singh P. Gupta O. P. Manas A. Gupta S. (2021). Role of molecular techniques in PMI estimation: an update. J. Forensic Leg. Med. 83, 102251. 10.1016/j.jflm.2021.102251 34592482

[B43] Sato T. Zaitsu K. Tsuboi K. Nomura M. Kusano M. Shima N. (2015). A preliminary study on postmortem interval estimation of suffocated rats by GC-MS/MS-based plasma metabolic profiling. Anal. Bioanal. Chem. 407 (13), 3659–3665. 10.1007/s00216-015-8584-7 25749795

[B44] Sharma V. Kumar R. (2018). Trends of chemometrics in bloodstain investigations. TrAC Trends Anal. Chem. 107, 181–195. 10.1016/j.trac.2018.08.006

[B45] Shen L. Ma J. Zhou H. Chen L. Tang J. Zhang K. (2022). Plasma metabolomic profiling reveals preliminary biomarkers of pork quality based on pH value. Foods 11 (24), 4005. 10.3390/foods11244005 36553747 PMC9778167

[B46] Sibbens L. Van de Voorde W. Decorte R. Bekaert B. (2017). The development of a forensic clock to determine time of death. Forensic Sci. Int. Genet. Suppl. Ser. 6, e162–e163. 10.1016/j.fsigss.2017.09.059

[B47] Szeremeta M. Pietrowska K. Niemcunowicz-Janica A. Kretowski A. Ciborowski M. (2021). Applications of metabolomics in forensic toxicology and forensic medicine. Int. J. Mol. Sci. 22 (6), 3010. 10.3390/ijms22063010 33809459 PMC8002074

[B48] Szeremeta M. Samczuk P. Pietrowska K. Kowalczyk T. Przeslaw K. Sieminska J. (2022). *In vitro* animal model for estimating the time since death with attention to early postmortem stage. Metabolites 13 (1), 26. 10.3390/metabo13010026 36676951 PMC9861157

[B49] Wang Q. He H. Li B. Lin H. Zhang Y. Zhang J. (2017). UV-Vis and ATR-FTIR spectroscopic investigations of postmortem interval based on the changes in rabbit plasma. PLoS One 12 (7), e0182161. 10.1371/journal.pone.0182161 28753641 PMC5533326

[B50] Wenzlow N. Mills D. Byrd J. Warren M. Long M. T. (2023). Review of the current and potential use of biological and molecular methods for the estimation of the postmortem interval in animals and humans. J. Vet. Diagn Investig. 35 (2), 97–108. 10.1177/10406387231153930 36744749 PMC9999395

[B51] Wilk L. S. Edelman G. J. Roos M. Clerkx M. Dijkman I. Melgar J. V. (2021). Individualised and non-contact post-mortem interval determination of human bodies using visible and thermal 3D imaging. Nat. Commun. 12 (1), 5997. 10.1038/s41467-021-26318-4 34650046 PMC8517003

[B52] Wilk L. S. Hoveling R. J. M. Edelman G. J. Hardy H. J. J. van Schouwen S. van Venrooij H. (2020). Reconstructing the time since death using noninvasive thermometry and numerical analysis. Sci. Adv. 6 (22), eaba4243. 10.1126/sciadv.aba4243 32523999 PMC7259946

[B53] Wilson S. J. Christensen A. M. (2017). A test of the citrate method of PMI estimation from skeletal remains. Forensic Sci. Int. 270, 70–75. 10.1016/j.forsciint.2016.11.026 27915189

[B54] Wu Z. Lu X. Chen F. Dai X. Ye Y. Yan Y. (2018). Estimation of early postmortem interval in rats by GC-MS-based metabolomics. Leg. Med. (Tokyo) 31, 42–48. 10.1016/j.legalmed.2017.12.014 29310000

[B55] Yumba-Mpanga A. Struck-Lewicka W. Wawrzyniak R. Markuszewski M. Roslan M. Kaliszan R. (2019). Metabolomic heterogeneity of urogenital tract cancers analyzed by complementary chromatographic techniques coupled with mass spectrometry. Curr. Med. Chem. 26 (1), 216–231. 10.2174/0929867324666171006150326 28990506

[B56] Zeki Ö. Eylem C. C. Reçber T. Kır S. Nemutlu E. (2020). Integration of GC-MS and LC-MS for untargeted metabolomics profiling. J. Pharm. Biomed. Anal. 190, 113509. 10.1016/j.jpba.2020.113509 32814263

[B57] Zelentsova E. A. Yanshole L. V. Melnikov A. D. Kudryavtsev I. S. Novoselov V. P. Tsentalovich Y. P. (2020). Post-mortem changes in metabolomic profiles of human serum, aqueous humor and vitreous humor. Metabolomics 16 (7), 80. 10.1007/s11306-020-01700-3 32613532

[B58] Zhang F. Y. Wang L. L. Zhang M. Dong W. W. Zhang Z. D. Li X. J. (2022a). Inferring postmortem submersion interval in rats found in water based on vitreous humor metabolites. Fa Yi Xue Za Zhi 38 (1), 59–66. 10.12116/j.issn.1004-5619.2021.410613 35725705

[B59] Zhang F. Y. Wang L. L. Dong W. W. Zhang M. Tash D. Li X. J. (2022b). A preliminary study on early postmortem submersion interval (PMSI) estimation and cause-of-death discrimination based on nontargeted metabolomics and machine learning algorithms. Int. J. Leg. Med. 136 (3), 941–954. 10.1007/s00414-022-02783-4 35099605

[B60] Zhang Y. Fan S. Wohlgemuth G. Fiehn O. (2023). Denoising autoencoder normalization for large-scale untargeted metabolomics by gas chromatography–mass spectrometry. Metabolites 13, 944. 10.3390/metabo13080944 37623887 PMC10456436

