# Supplementary material for: Comparative analysis of anticoagulant influence on PMI estimation based on porcine blood metabolomics profile measured using GC-MS
Source: Front Mol Biosci. 2025 Jan 7;11:1400622. doi: 10.3389/fmolb.2024.1400622 (PMC11746058; doi:10.3389/fmolb.2024.1400622)
Supplement: Supplementary file 1 [file DataSheet1.pdf]

Supplementary materials to:

Comparative analysis of anticoagulant influence on PMI estimation based on porcine blood metabolomics profile measured using GC-MS

Patrycja Mojsak[1], Paulina Samczuk[1,2], Paulina Klimaszewska[1], Michal Burdukiewicz[1,3], Jaroslaw Chilimoniuk[1], Krystyna Grzesiak[1,4], Karolina Pietrowska[1], Justyna Ciborowska[5], Anna Niemcunowicz–Janica[6], Adam Kretowski[1,7], Michal Ciborowski[1,8], Michal Szeremeta[6]

[1]Metabolomics and Proteomics Laboratory, Clinical Research Centre, Medical University of Bialystok, Bialystok, Poland.

[2]Department of Genetic Research, Central Forensic Laboratory of the Police, Warsaw, Poland.

[3]Institute of Biotechnology and Biomedicine, Autonomous University of Barcelona, Cerdanyola del Vallès, Spain.

[4]Faculty of Mathematics and Computer Science, University of Wroclaw, Wroclaw, Poland.

[5]Chemical Research Laboratory, Forensic Laboratory of the Voivodeship Police Headquarters in Bialystok, Bialystok, Poland.

[6]Department of Forensic Medicine, Medical University of Bialystok, Bialystok, Poland.

[7]Department of Endocrinology, Diabetology and Internal Medicine, Medical University of Bialystok, Bialystok, Poland.

[8]Department of Medical Biochemistry, Medical University of Bialystok, Bialystok, Poland.

Figure S1. Statistically significant metabolites depending on time and the addition of anticoagulant: Panel a) - a figure taking into account the biodiversity of the tested pigs, panel b) - a figure taking into account the median of all id for each time, metabolite and EDTA + q1 and q3.

Creatinine

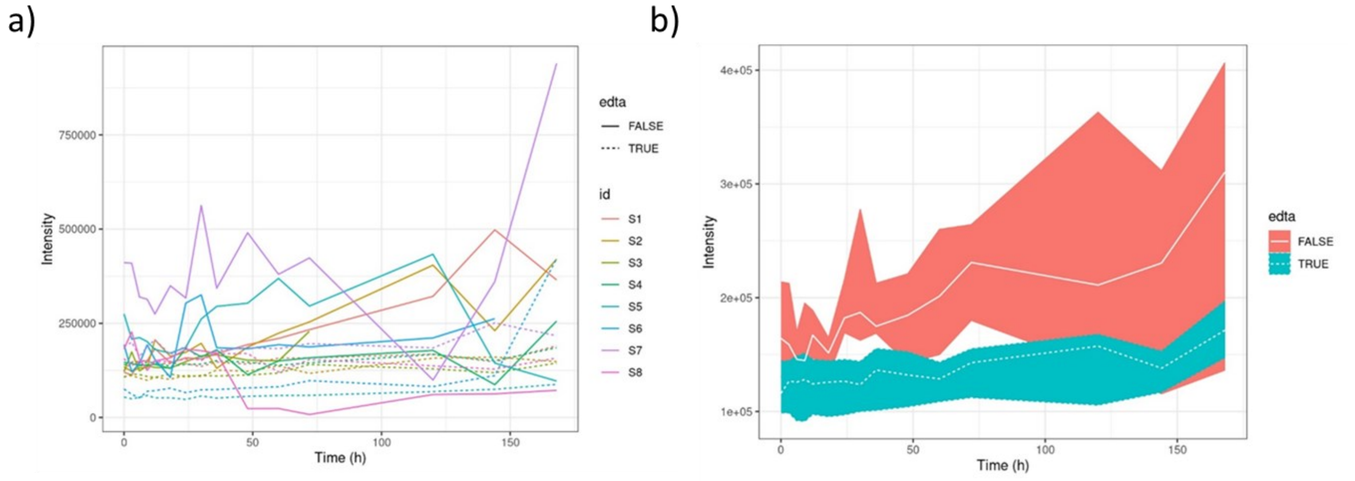

Iminodiacetic acid

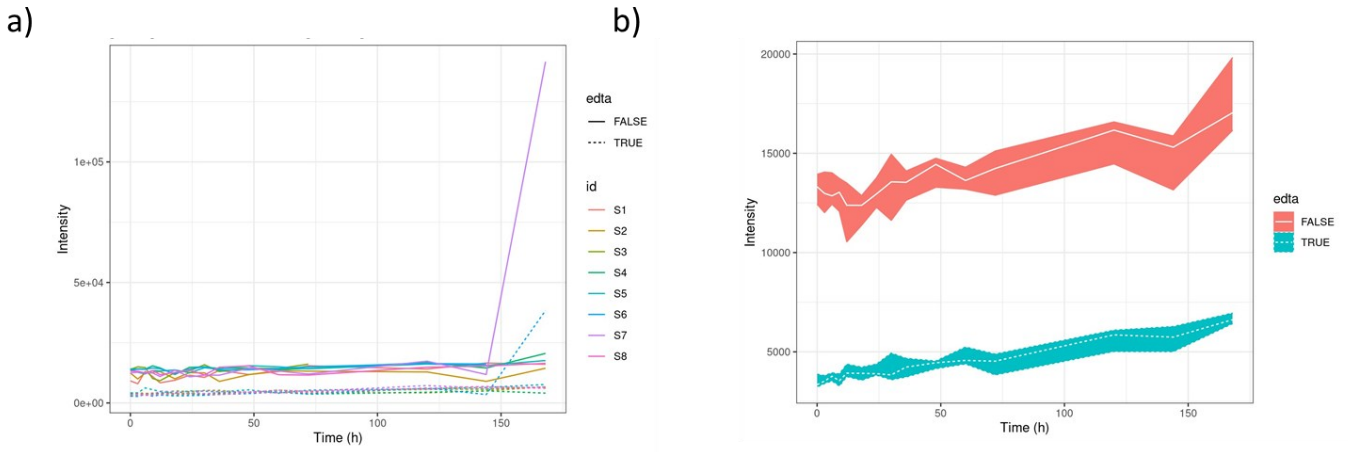

Isoleucine

a)

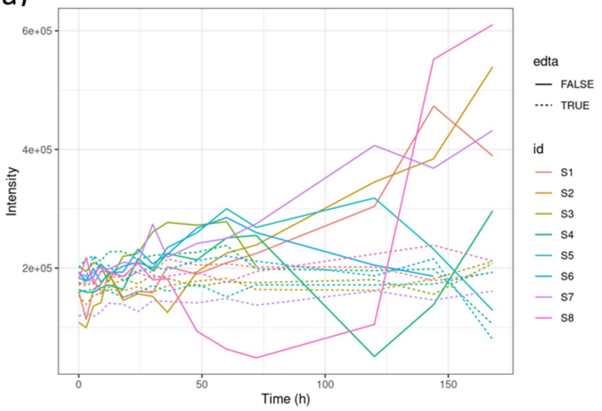

b)

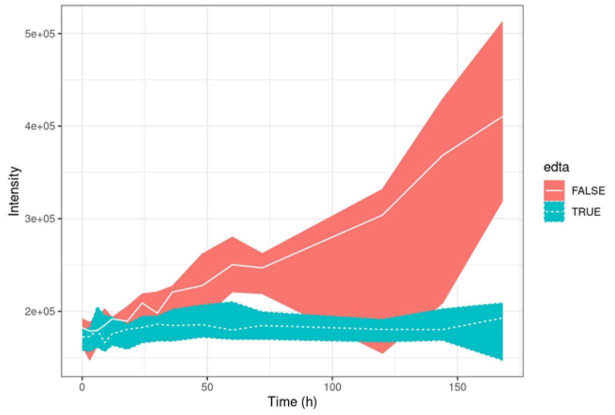

Lysine

a)

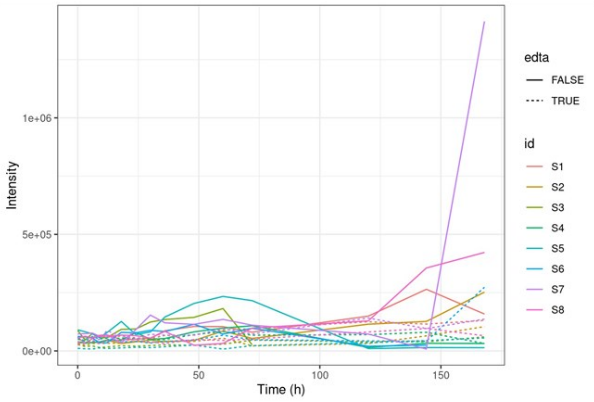

b)

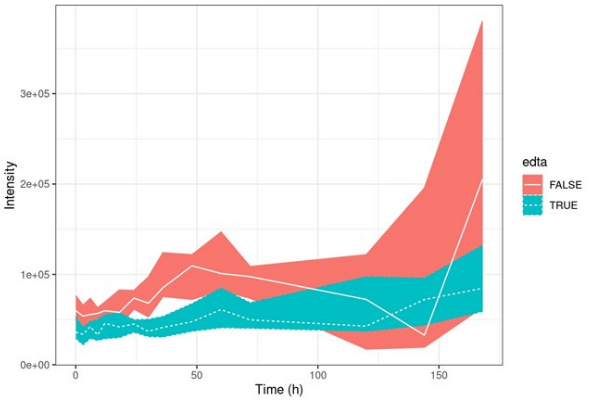

Ornithine

a)

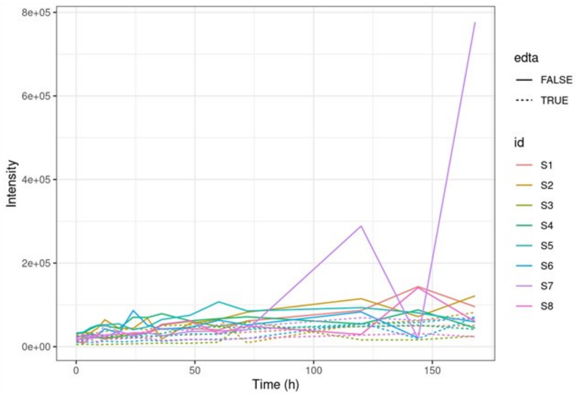

b)

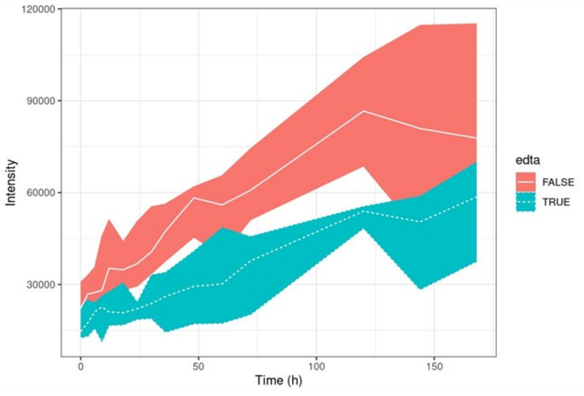

Phenylalanine

a)

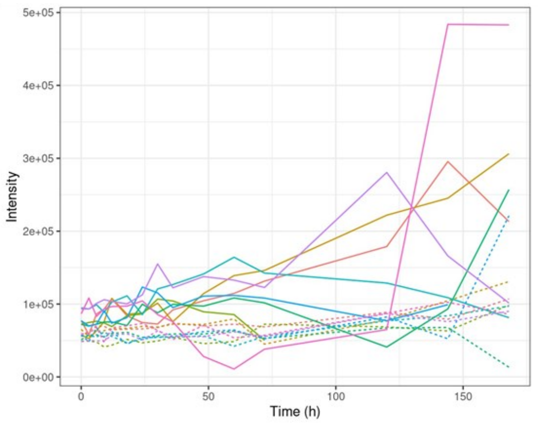

b)

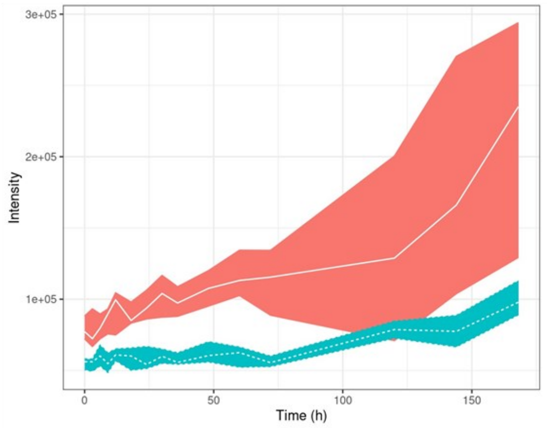

Threonine

a)

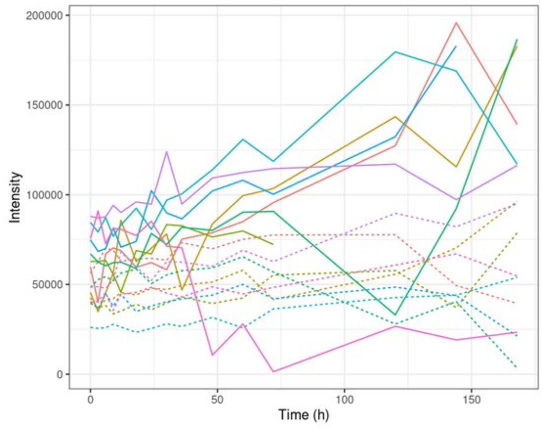

b)

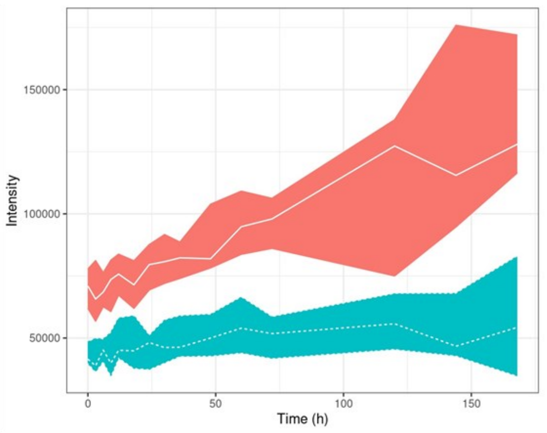

Valine

a)

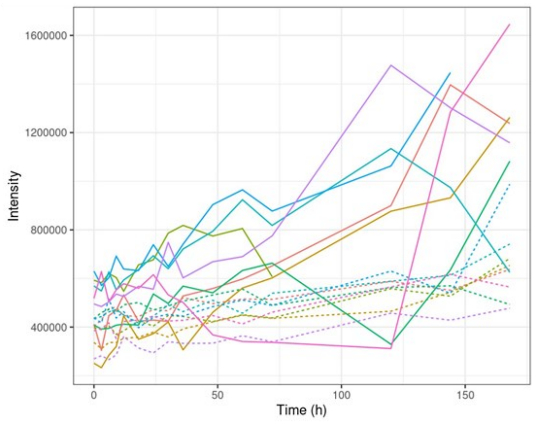

b)

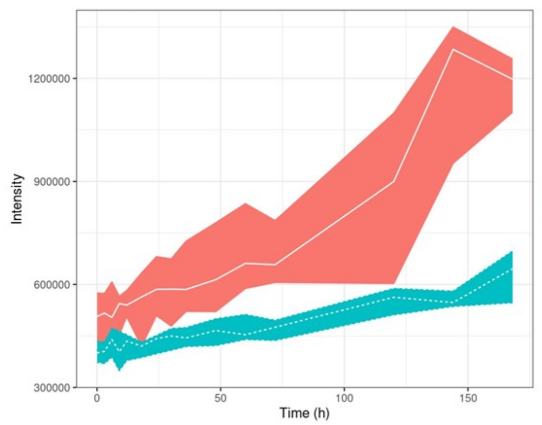

Pyroglutamic acid

a)

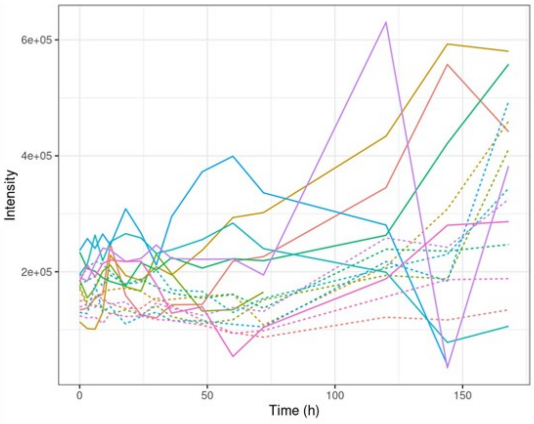

b)

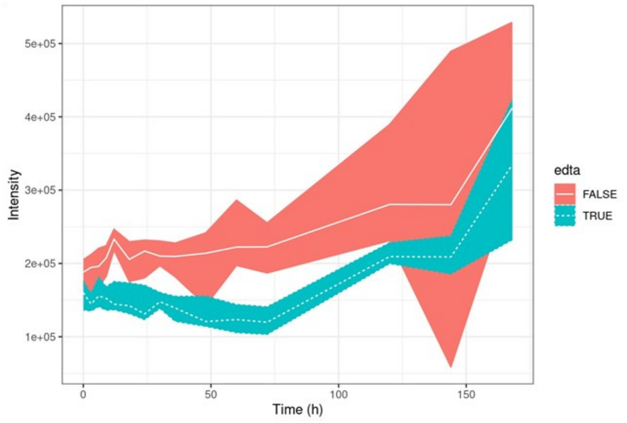

Pyruvic acid

a)

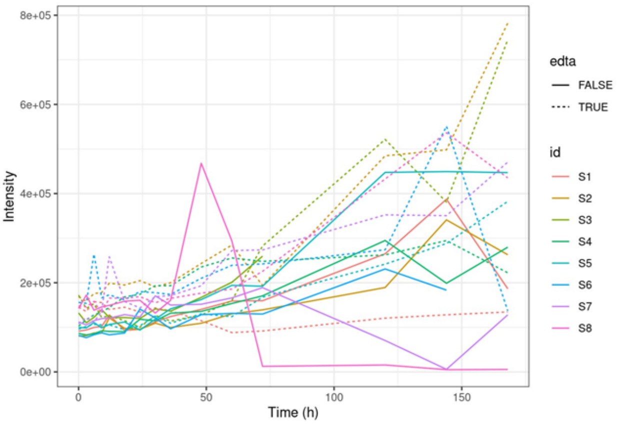

b)

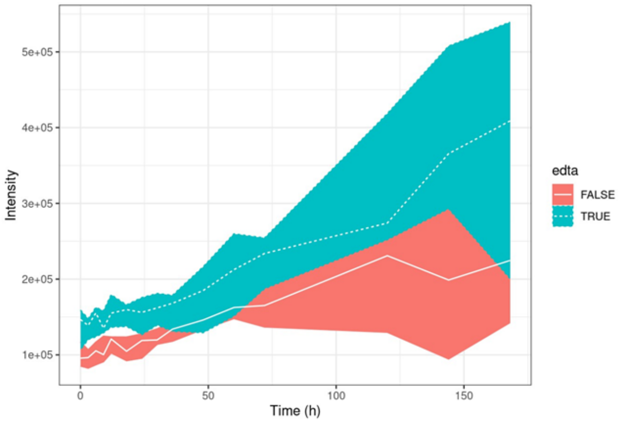

Pyranose (Glucose/Mannose)

a)

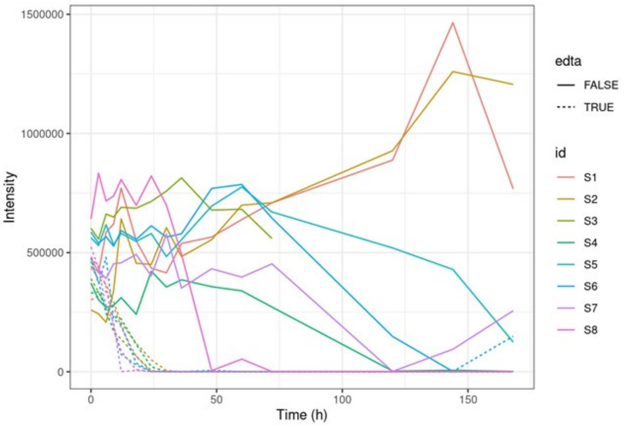

b)

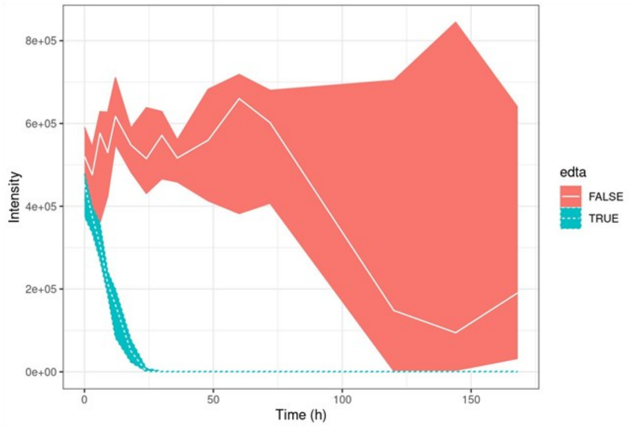

1,5 – anhydro-D-sorbitol

a)

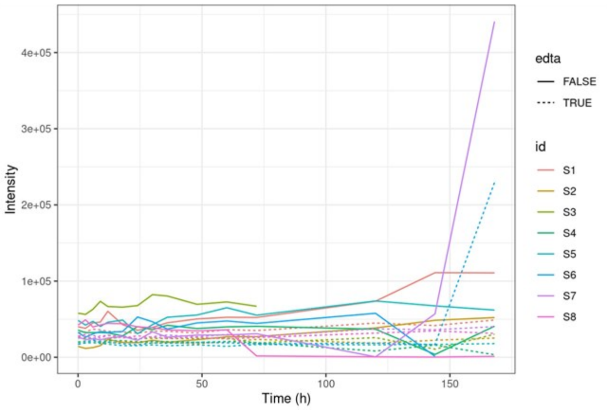

b)

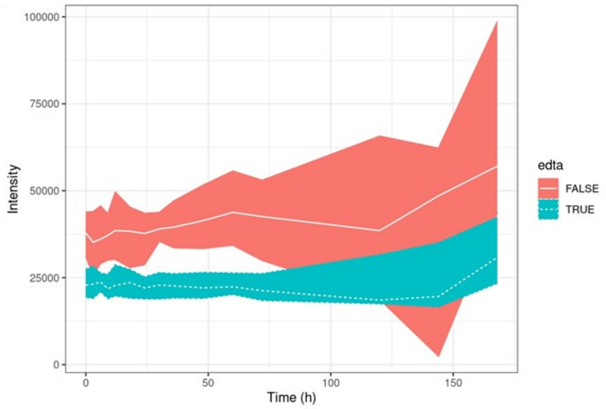

Fumaric acid

a)

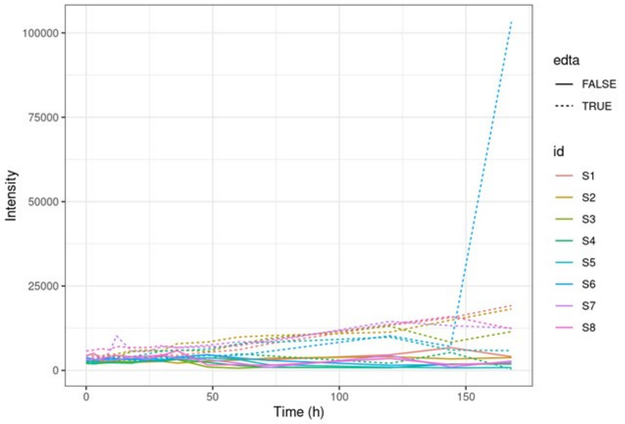

b)

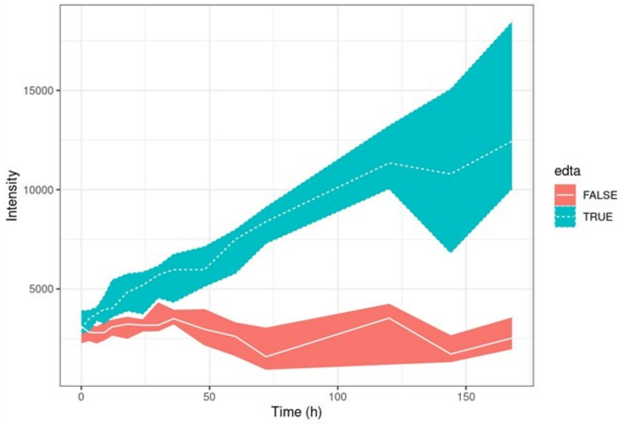

Glycerol-1-phosphate

a)

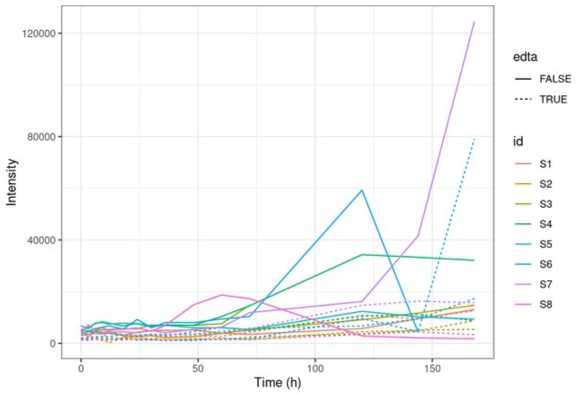

b)

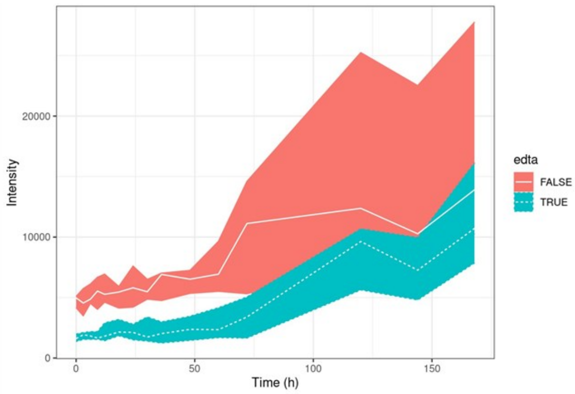

Hypoxanthine

a)

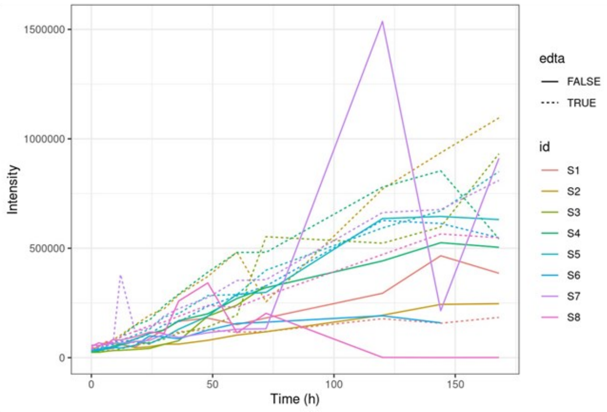

b)

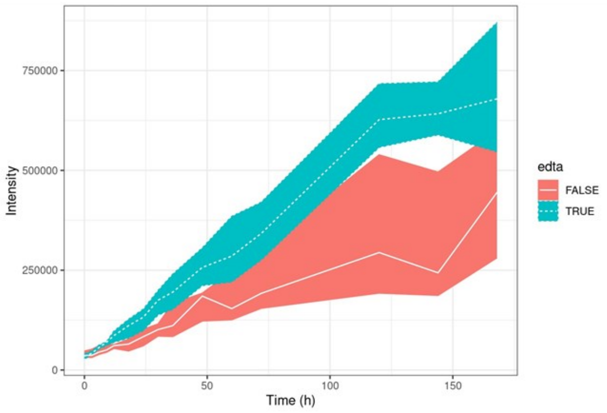

Table S1. List of identified metabolites. (RT, retention time; HMDB - Human Metabolome Database; TI, target ion; QI 1, first qualifier ion; QII 2, second qualifier ion; RI, retention index. All compounds reported in the table are three methyl silylated (TMS), and the following number refers to the number of active hydrogen replaced with the TMS group, as reported in the Fiehn library.

	Metabolites	Group of metabolites	HMDB	RT	TI	QI 1	QII 2	RI
1	Boric acid 3TMS	Miscellaneous metallic oxoanionic compounds	HMDB35731	6.3	221	263	73	704
2	Pyruvic acid	Alpha-keto acids and derivatives	HMDB00243	6.58	174	89	59	724
3	Lactic acid	Alpha hydroxy acids and derivatives	HMDB00190	6.76	117	147	73	733
4	Glycolic acid	Alpha hydroxy acids and derivatives	HMDB00115	6.96	177	147	73	747
5	Valine 1TMS	AAs, peptides, and analogues	HMDB00883	7.2	72	55	75	764
6	Valine 2TMS			9.15	144	218	73	893
7	3-methyl-2-oxobutanoic acid 2TMS	FA esters	HMDB30027	7.42	202	186	73	806
8	Butanoic acid, 2-(methoxyimino)-3-methyl-, trimethylsilyl ester	-	-	7.43	186	89	73	810
9	Alanine 1TMS	AAs, peptides, and analogues	HMDB00161	7.47	116	190	73	777
10	2-ketobutyric acid	Short-chain keto acids and derivatives	HMDB00005	7.5	188	129	89	779
11	2-hydroxybutyric acid	Alpha hydroxy acids and derivatives	HMDB00008	7.68	131	205	73	800
12	Oxalic acid	Dicarboxylic acids and derivatives	HMDB02329	7.75	190	147	73	802
13	(R)-3-Hydroxybutyric acid, trimethylsilyl ether, trimethylsilyl ester	Beta hydroxy acids and derivatives	HMDB00011	8.18	233	191	117	815
14	Pentanoic acid, 2-[(trimethylsilyl)oxy]-, trimethylsilyl ester	-	-	8.24	145	103	73	830
15	2-hydroxy-3-methylbutyric acid,	Fatty acids and conjugates	HMDB00407	8.24	219	145	73	830

	2TMS							
16	Pentanoic acid, 2-(methoxyimino)-3-methyl-, trimethylsilyl ester	-	-	8.43	200	189	73	849
17	2-ketoisocaproic acid 1TMS	Short-chain keto acids and derivatives	HMDB00695	8.44	189	200	73	850
18	2-ketoisocaproic acid 2TMS			8.92	200	216	73	885
19	2-keto-3-methylvaleric acid mo-tms pk2	Short-chain keto acids and derivatives	HMDB00491	8.72	290	91	73	872
20	Benzoic acid	Benzoic acids and derivatives	HMDB01870	9.47	179	105	135	919
21	Urea	Ureas	HMDB00294	9.59	189	147	73	920
22	Serine 1TMS	AAs, peptides, and analogues	HMDB00187	9.6	116	132	73	928
23	Serine 2TMS			10.96	204	218	73	1023
24	Phosphoric acid	Non-metal phosphates	HMDB01429	9.77	299	211	133	948
25	Ethanolamine	Amines	HMDB00149	9.87	174	100	147	941
26	Isoleucine 2TMS	AAs, peptides, and analogues	HMDB00172	10.05	158	218	73	968
27	Proline 2TMS	AAs, peptides, and analogues	HMDB00162	10.14	142	216	73	975
28	Glycine	AAs, peptides, and analogues	HMDB00123	10.26	174	248	73	985
29	Succinic acid	Dicarboxylic acids and derivatives	HMDB00254	10.34	247	129	147	989
30	Glyceric acid	CARBs and CARB conjugates	HMDB00139	10.73	292	189	73	986
31	Citraconic acid	Fatty acids and conjugates	HMDB00634	10.79	241	255	99	991
32	Fumaric acid	Dicarboxylic acids and derivatives	HMDB00134	10.94	245	147	73	1003
33	Pelargonic acid (nonanoic acid)	FAs and conjugates	HMDB00847	11	215	117	129	1019
34	Threonine 2TMS	AAs, peptides, and analogues	HMDB00167	11.3	218	291	117	1048
35	Beta-alanine 1TMS	Amino acids, peptides, and analogues	HMDB00056	11.87	290	248	174	1097
36	Aminomalonic acid, tris(trimethylsilyl)-	AAs, peptides, and analogues	HMDB01147	12.39	320	218	73	1141
37	Malic acid	Beta hydroxy acids and derivatives	HMDB31518	12.61	233	245	147	1161
38	Trans-4-hydroxy-L-proline 1TMS	AAs, peptides, and analogues	HMDB00725	12.61	158	260	68	1146
39	Threitol 4TMS	CARB and CARB conjugates	HMDB04136	12.63	217	205	103	1175
40	Methionine 2TMS	AAs, peptides, and analogues	HMDB00696	13	176	219	128	1195
41	5-Oxoproline 2TMS	AAs, peptides, and analogues	HMDB00267	13.05	156	230	258	1197

42	Iminodiacetic acid 2TMS	AAs, peptides, and analogues	HMDB11753	13.28	232	306	73	1183
43	Threonic acid	CARBs and CARB conjugates	HMDB00943	13.3	292	205	220	1217
44	Glutamic acid 1TMS	AAs, peptides, and analogues	HMDB00148	13.34	174	276	84	1188
45	Glutamic acid 2TMS			14.15	246	128	73	1287
46	Creatinine	AAs, peptides, and analogues	HMDB00562	13.63	115	329	314	1215
47	Alpha ketoglutaric acid	Keto acids and derivatives	HMDB00208	13.86	198	288	73	1237
48	Hypotaurine	Sulfinic acids	HMDB00965	14.11	188	100	73	1260
49	Phenylalanine 2TMS	AAs, peptides, and analogues	HMDB00159	14.24	218	192	73	1290
50	Taurine	Organosulfonic acids and derivatives	HMDB00251	14.94	326	174	73	1338
51	Monoamidomalonic acid, tris(trimethylsilyl)	-	-	15.25	214	304	73	1430
52	Ornithine 3TMS	AAs, peptides, and analogues	HMDB00214	15.75	142	348	73	1488
53	Glycerol 1-phosphate	Glycerophosphates	HMDB00126	15.85	299	357	73	1428
54	Citric acid	Tricarboxylic acids and derivatives	HMDB00094	16.33	273	347	147	1486
55	Hypoxanthine	Purines and purine derivatives	HMDB00157	16.48	265	280	206	1472
56	1,5-anhydro-D-sorbitol	CARBs and CARB conjugates	HMDB02712	16.97	217	191	129	1522
57	Mannose 1TMS	CARBs and CARB conjugates	HMDB00169	17.13	319	205	147	1555
58	Mannose 2TMS			17.45	205	319	147	1570
59	Tyrosine 1TMS	AAs, peptides, and analogues	HMDB00158	17.19	179	208	73	1562
60	Tyrosine 2TMS			17.86	218	280	73	1597
61	Glucose 1TMS	CARBs and CARB conjugates	HMDB00122	17.25	319	205	147	1569
62	Lysine 2TMS	AAs, peptides, and analogues	HMDB00182	17.53	174	317	156	1592
63	Mannitol	Carbohydrates and carbohydrate conjugates	HMDB00765	17.67	319	205	73	1592
64	Gluconic acid 2TMS	Carbohydrates and carbohydrate conjugates	HMDB00625	18	333	292	205	1646
65	Scyllo-Inositol 6TMS	Alcohols and polyols	HMDB06088	18.56	318	217	191	1691
66	Palmitoleic acid	FAs and conjugates	HMDB03229	18.58	311	117	129	1695
67	Palmitic acid	FAs and conjugates	HMDB00220	18.85	313	117	129	1708

68	Isopropyl beta-D-1-thiogalactopyranoside	Carbohydrates and carbohydrate conjugates	HMDB0253575	19.09	361	217	73	1736
69	Myo-Inositol 6TMS	Alcohols and polyols	HMDB00211	19.18	217	305	318	1765
70	Heptadecanoic acid	Fatty acids and conjugates	HMDB02259	19.72	327	117	73	1800
71	Tryptophan 2TMS	Indolyl carboxylic acids and derivatives	HMDB00929	20.23	202	291	73	1881
72	Oleic acid	FAs and conjugates	HMDB00207	20.36	339	117	129	1886
73	Linoleic acid	Lineolic acids and derivatives	HMDB00673	20.4	337	129	262	1873
74	Trans-13-octadecenoic acid	FAs and conjugates	HMDB41480	20.43	339	117	129	1899
75	Stearic acid	FAs and conjugates	HMDB00827	20.59	341	117	129	1907
76	Cystine 2TMS	Amino acids, peptides, and analogues	HMDB00192	20.77	339	218	73	1929
77	Cystine 3TMS			21.1	411	218	73	1959
78	5-hydroxy-L-tryptophan 1TMS	Tryptamines and derivatives	HMDB00472	22	290	218	73	2087
79	Cholesterol	Cholestane steroids	HMDB00067	27.48	329	129	368	2878

(RT, retention time; HMDB - Human Metabolome Database; TI, target ion; QI 1, first qualifier ion; QII 2, second qualifier ion; RI, retention index. All compounds reported in the table are three methyl silylated (TMS), and the following number refers to the number of active hydrogen replaced with the TMS group, as reported in the Fiehn library.
